# Skin prick test in milk allergic patients undergoing oral immunotherapy: Does the milk form used for skin tests matter?

**DOI:** 10.3389/falgy.2022.974626

**Published:** 2022-08-08

**Authors:** Esraa Bukhari, Sofianne Gabrielli, Christine McCusker, Julia Upton, Eyal Grunebaum, Edmond S. Chan, Liane Beaudette, Alexandra Langlois, Bahar Torabi, Duncan Lejtenyi, Ann E. Clarke, Danbing Ke, Bruce David Mazer, Moshe Ben-Shoshan

**Affiliations:** ^1^ Division of Allergy and Clinical Immunology, Department of Pediatrics, Montreal Children’s Hospital, McGill University Health Centre, Montreal, QC, Canada; ^2^ Department of Pediatrics, Faculty of Medicine, King Abdulaziz University, Jeddah, Saudi Arabia; ^3^ Division of Immunology and Allergy, Department of Pediatrics, The hospital for Sick Children, University of Toronto, Toronto, ON, Canada; ^4^ Division of Allergy and Immunology, Department of Pediatrics, BC Children’s Hospital, University of British Columbia, Vancouver, BC, Canada; ^5^ Division of Allergy and Clinical Immunology, Department of Pediatrics, Centre de recherche du centre hospitalier universitaire de Sherbrooke, University of Sherbrooke, Sherbrooke, QC, Canada; ^6^ Division of Rheumatology, Department of Medicine, Cumming School of Medicine, University of Calgary, Calgary, AB, Canada; ^7^ The Research Institute of the McGill University Health Centre, Division of Pediatric Allergy and Clinical Immunology, Department of Pediatrics. Montreal Children’s Hospital, Montreal, QC, Canada

**Keywords:** milk, allergy, oral immunotherapy (OIT), Desensitization, skin prick test (SPT), diluted milk, extract

## Abstract

SPT is the most commonly used confirmatory test for an IgE-mediated milk allergy. However, food SPTs are not standardized. We aimed to assess the accuracy of SPTs with extract, diluted, and undiluted milk to detect desensitization in children with milk allergy undergoing OIT. Children with milk allergy undergoing OIT and controls were recruited from Montreal Children’s Hospital (MCH), British Columbia Children’s Hospital (BCCH) and The Hospital for Sick Children (SickKids). Participants in the active arm received a weekly increase in milk until 200 ml of pure milk was tolerated. SPT using milk extract (Omega), diluted 2% milk (1:10), and undiluted milk was done at the study entry and when 200 ml of pure milk was reached. Participants in the control arm had SPT at study entry and 12 months later before they entered the active arm. Among 53 children who reached 200 ml, the median age was 12 years and 54.7% were males. The mean decrease in wheal size at 200 ml from the baseline was 3.78 mm (95%CI, 2.55–5.01), 5.05 mm (95% CI, 3.68–6.41), and 5.05 mm (95% CI, 3.29–6.80) for milk extract, diluted and undiluted milk respectively. Among 32 controls, the median age was 10 years and 62.5% were males. There was no significant change in wheal diameter over a one-year period regardless of the skin test method. Response to extract behaved similarly to whole food (Diluted and undiluted) and thus can be used to follow sensitization in the context of a desensitization program.

## Introduction

Foods are a common cause of anaphylaxis and account for the majority of fatal reactions in children (1). Cow’s milk allergy (CMA) is a common and well recognized food allergy in infants and young children (2). The prevalence of milk allergy is reported to range between 3% to 17% (3). Among all Canadians, 2.6% self-report milk allergy (4). The majority of patients present between 6 and 12 months of life, when milk is considered a major nutritional source and strict avoidance could lead to nutritional deficiencies if not substituted properly by appropriate alternatives.

The diagnosis of milk-induced IgE-mediated reactions is based on corroboration of clinical history and confirmatory tests. A skin prick test (SPT) is the most commonly used confirmatory tests among allergists due to high accessibility and ease of use and is often used as the only test to assess outgrowing milk allergy and determine if a food challenge should be conducted. However, food-related SPTs in general and, in particular milk, are not standardized. Further it is not clear if SPTs with pure/diluted milk may be more accurate for the diagnosis of temporal changes in milk allergy. We aimed to evaluate temporal changes in SPT diameter for milk extract, diluted milk, and undiluted milk for all participants who underwent OIT when 200 ml of cow’s milk was reached and to compare changes in SPT diameter in children who underwent desensitization versus controls.

## Methods

A randomized controlled trial with crossover design was conducted at three different Canadian hospitals: Montreal Children’s Hospital (MCH), British Columbia Children’s Hospital (BCCH), and the Hospital for Sick Children (SickKids) clinical investigation units from July 2013 until November 2020.

At study entry, all participants underwent a controlled single-blinded oral challenge. Only patients with a positive challenge were included and were randomized to either the oral immunotherapy (OIT) or control group. After one year, participants in the control arm were offered OIT which was started with another blinded oral challenge. All controls were asked to repeat skin tests when entering the active arm. The desensitization protocol was previously described (5). SPTs using milk extract (Omega, Port Washington, NY), diluted 2% milk (1:10), and undiluted 2% pasteurized milk were performed at the study entry, and when 200 ml of 2% undiluted milk dose was reached using the same preparation and same day milk dilution. All statistical analyses were performed using R version 4.0.0 (R Core Team [2013] R: A language and environment for statistical computing Foundation for Statistical Computing, Vienna, Austria). Descriptive statistics of the variables included mean with standard deviation (SD) or median with interquartile range (IQR). Paired t-tests were performed to compare the SPT results at study entry to the endpoint (200 mL dose) and for the control at the study entry and after one-year observation.

A *p*-value of <0.05 was considered significant. Linear regression models were used to assess factors associated with a decrease in SPT diameter (Table 3).

This study was approved by the Research Ethics Committee of the MCH, BCCH, and SickKids. Written informed consent was obtained after the explanation of all the study processes and phases.

## Results

**Figure 1:**
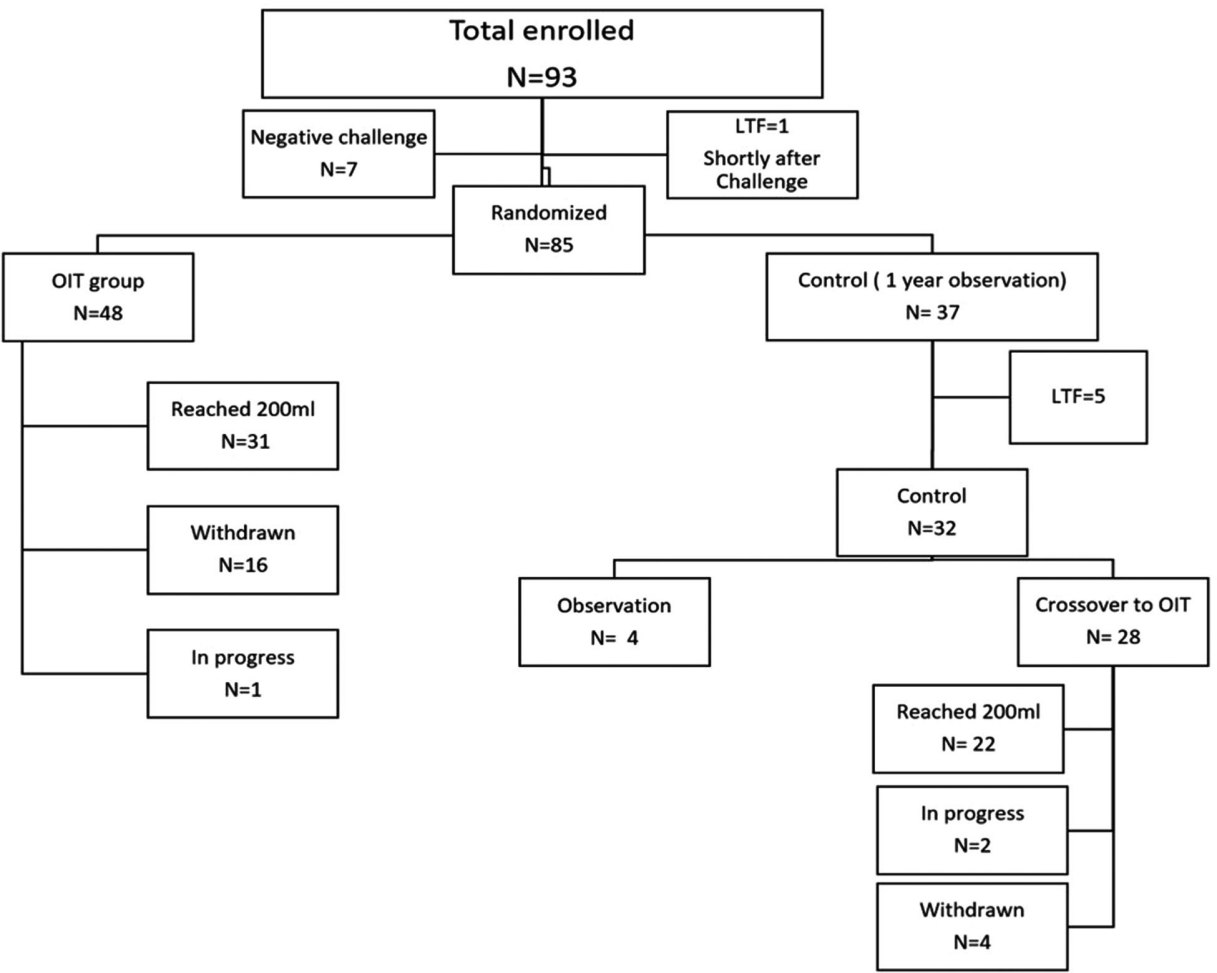
Total of 93 participants were recruited for this study, 8 children were excluded (7 passed the entry challenge and 1 lost to follow-up shortly after the challenge). The remaining 85 children were randomized to either OIT (*n* = 48) or control (*n* = 37). Among the 48 children in the OIT group, 16 were withdrawn and 31 children reached 200 ml of pure milk. Among the 37 controls, 28 children crossed over to OIT after one year of observation, 5 LTF and the remaining 4 are still in the observation period. Out of the 28 who crossed over to OIT, 22 children reached 200 ml. *LTF, Lost to follow; OIT, Oral Immunotherapy.

In this study, 93 participants were recruited, 8 children were excluded (7 passed the entry challenge and 1 lost to follow-up shortly after the challenge. A total of 85 participants were eligible and randomized to either OIT (*n* = 48) or control (*n* = 37) (Figure 1). After one year of observation, 28 children in the control group crossed-over to OIT, of which 22 children reached 200 ml. In the current analysis, we focused on the 53 children who reached 200 ml of pure milk (OIT, *n* = 31 and crossed-over control, *n* = 22). Among 53 children who reached 200 ml, the median age was 12 years [IQR 9.00, 15.00)], 54.7% (*n* = 29) were males, 35.8% (*n* = 19) had active eczema and 75.4% (*n* = 40) had controlled asthma (Table 1). The mean decrease in wheal size at 200 ml from baseline was 3.78 mm (95%CI, 2.55–5.01, *p*-value = 2.231 × 10^−8^) for the milk extract, 5.05 mm (95% CI, 3.68–6.41, *p*-value = 7.61 × 10^−11^) for the diluted milk and 5.05 mm (95% CI, 3.29–6.80, *p*-value = 1.165 × 10^−7^) for the undiluted milk (Table 2). Among 32 controls, the median age was 10 years (IQR 7.0, 14.25) and 62.5% (*n* = 20) were males, 37.5% (*n* = 12) had active eczema and 75% (*n* = 24) had controlled asthma (Table 1). There was no significant change in wheal diameter over one year, regardless of the skin test method (Table 2).

**Table 1:** Demographic data among children undergoing OIT.

	Control prior to OIT	Reached 200 ml
Total No.	32	53
Reached 200 ml, *n* (%)	22 (68.8%)	53
Median age at challenge in years	10 (7.0, 14.25)	12.0 (9.00,15.00)
Age at diagnosis in months, median (IQR)	8 (4.00,10.0)	6 (4.00,10.0)
Male gender, *n* (%)	20 (62.5%)	29 (54.7%)
Active eczema, *n* (%)	12 (37.5%)	19 (35.8%)
Resolved eczema, *n* (%)	5 (15.6%)	7 (13.2%)
Controlled Asthma, *n* (%)	24 (75%)	40 (75.4%)
Resolved Asthma, *n* (%)	0	2 (3.77%)
Pollen Allergy, *n* (%)	20 (62.5%)	25 (47.2%)
Other food allergy, *n* (%)	5 (15.6%)	13 (24.5%)

OIT, Oral immunotherapy; IQR, Interquartile Range.

**Table 2:** SPT for children who have reached 200 ml endpoint vs. control group one-year after study entrance.

	SPT for Children who have Reached 200 ml Endpoint					SPT for Control Group One-Year after Study Entrance				
	SPT baseline in mm Mean (SD)	SPT at 200 ml in mm Mean (SD)	Difference	95% CI	P value	SPT at baseline in mm mean (SD)	SPT at 1-year post-entrance in mm, mean (SD)	Difference	95% CI	*p*-value
Extract	7.23 (3.55)	3.45 (2.74)	3.78	2.55-5.01	2.231 × 10^−8^	7.89 (3.84)	6.46 (3.49)	1.43	−0.84–3.34	0.2362
Diluted	8.28 (4.07)	3.23 (2.9)	5.05	3.68-6.41	7.61 × 10^−11^	7.83 (4.01)	7.34 (4.4)	0.49	−0.97–3.45	0.2659
Milk (pure 2%)	11.15 (4.93)	6.10 (4.14)	5.05	3.29-6.80	1.165 × 10^−7^	11.35 (6.13)	9.7 (4.68)	1.65	−1.24–4.8	0.2386

SPT, Skin Prick Test.

For those who passed the initial challenge and were excluded for the desensitization: The mean SPT was 3.1 ± 2.2 (range 0–6) for the extract, 3 ± 1.7 (1–6) for the diluted, and 6 ± 4.4 (1–13) for the undiluted.

Wilcoxon test was done to assess the difference in the baseline SPT for the participant who passed the initial challenge and the participant who entered the study (who reached 200 ml and those who didn’t) and there was a significant difference for all milk forms used in SPT with *p*-value of 0.001408 for the extract, 0.0008837 for the diluted and 0.009871 for the undiluted.

For the participants who failed to reach the full 200 ml, the mean SPT at baseline was 8.9 ± 5.1(2–25) for the extract, 8.2 ± 4.9(0–20) for the diluted, and 12.5 ± 6.1(3–26) for 2% undiluted milk. Compared to those who reached 200 ml the mean SPT at baseline was 7.23(0–17) for the extract, 8.28 for the diluted (0–20), and 11.15(4–32) for 2% undiluted milk.

Wilcoxon test was done to assess the difference in the baseline SPT for the participant who failed to reach 200 ml and the participant who reached 200 ml and there was no significant difference with *p*-value of 0.2475 for the extract, 0.8673 for the diluted and 0.3368 for the undiluted. A multivariable linear regression model was used to examine factors associated with the difference in SPT diameter (Table 3). The decrease in the undiluted milk SPT diameter was higher (by 4.38 mm) in patients with pollen allergy compared to patients with no history of pollen allergy (*p*-value = 0.01983). A larger SPT at baseline was associated with a larger decrease at 200 ml for undiluted milk (*p*-value =1.926 × 10^−10^), for diluted milk (*p*-value = 3.464 × 10^−10^), for milk extract and (*p*-value = 1.818 × 10^−10^).

**Table 3:** Variables included in the linear regression.

Variable	Beta-coefficient			95% Confidence Interval		
	Milk (2%)	Diluted	Undiluted	Milk (2%)	Diluted	Undiluted
Sex (Male)	1.139	0.1907	1.5277	−2.06, 2.44	−2.30, 2.41	−0.84, 3.90
Age at diagnosis	−0.007675	−0.08384	0.03355	−0.39, 0.38	−0.33, 0.16	−0.23, 0.30
Age at challenge	0.1075	−0.02481	−0.1974	−0.40, 0.61	−0.34, 0.29	−0.53, 0.14
Controlled Asthma	3.081	1.648	0.4987	−1.27, 7.67	−1.27, 4.57	−2.66, 3.66
Active Eczema	−1.883	−0.08842	−0.8400	−5.90, 2.13	−2.42, 2.25	−3.43, 1.75
Pollen Allergy	4.380*	−0.3022	0.2327	0.72, 8.03*	−2.76, 2.15	−2.35, 2.82
Other food Allergy	−4.258	−1.4816	−0.597	−8.39, -0.12	−4.08, 1.11	−3.69, 2.50
Moderate to severe reaction at entry challenge	0.6382	1.927	0.6928	−2.96, 4.23	−0.28, 4.14	−1.74, 3.12
Cumulative amount of milk at entry challenge	−0.009447	0.02853	−0.03306	−0.06, 0.04	−0.05, 0.00	−0.07, 0.00
SPT diameter at baseline	0.9435*	0.71490*	0.8796*	0.71, 1.18*	0.53, 0.90*	0.66, 1.1*

* Statistically significant. SPT, Skin Prick Test.

## Discussion

Previous studies assessed the use of SPT to diagnose milk allergy (6). Another retrospective study mentioned the role of SPT using commercial extract (Alk-Abello®, Horsholm, Denmark) and fresh milk in predicting the resolution of CMA in young children. SPT using fresh milk (prick to prick test) was the most remarkable predictor of unbaked milk tolerance (7). Babaie et al. used milk SPT (milk extract, whole milk, and serial dilutions of the whole milk) to identify the starting dilution for desensitization. SPT using milk extract was significantly reduced when 120 ml of milk was reached with the mean SPT of 12 ± 5.1 at the beginning and 5.83 ± 3.34 at the end of the desensitization (8).To our knowledge, no studies compared the accuracy of SPT using extract, diluted, or undiluted milk to detect desensitization in children undergoing OIT.

Our study showed that the majority of children in our cohort (62%) were successfully desensitized to milk. In addition, when comparing the accuracy of SPT using extract, diluted and undiluted milk to detect desensitization in children undergoing OIT we found that there was a significant decrease in SPT diameter with all forms of milk used for SPT in patients who reached 200 ml of pure milk and the difference reflected desensitization rather than time effect.

Wheal and flare reactions in SPT occur due to the IgE- mediated histamine release from activated mast cells and basophils. Desensitization appears to deviate the immune response away from IgE production (9). Decreased IgE production could account for the greater decrease in SPT diameter in patients with higher SPT at study entry.

Given that there was no significant difference in wheal diameter amongst controls who waited one year before entering the OIT group, the changes observed are unlikely due to the natural decrease in SPT diameter over time. Due to the small sample size in our study, we could not account for other factors associated with a decrease in SPT diameter.

Considering that our findings suggest that temporal changes in SPT reflect the success of desensitization, we would recommend consistently using the same form of extract or milk to assess SPT change over time.

In conclusion, our study establishes that SPT using milk (omega) extract is as accurate as undiluted and diluted milk in predicting milk desensitization in children. Therefore, clinicians can use the most available and easily accessible milk allergen for following milk desensitization in areas where commercial extract is not easily accessible. This observation applied only to the omega extract which might not apply to other commercial extracts as they haven’t been used in the current study.

## Data Availability

The raw data supporting the conclusions of this article will be made available by the authors, without undue reservation.
